# Unraveling the link: environmental tobacco smoke exposure and its impact on infertility among American women (18–50 years)

**DOI:** 10.3389/fpubh.2024.1358290

**Published:** 2024-03-08

**Authors:** Liang Peng, Xiaohan Luo, Baodi Cao, Xiaohui Wang

**Affiliations:** The Second People's Hospital of Jingdezhen, Jingdezhen, China

**Keywords:** environmental tobacco smoke, infertility, serum cotinine, non-linearity, NHANES

## Abstract

**Purpose:**

The detrimental effects of environmental tobacco smoke (ETS) on women’s reproductive health have been widely recognized. However, the detailed association between exposure to environmental tobacco smoke and the incidence of infertility remains under-explored. This investigation focuses on exploring this potential connection.

**Methods:**

For this analysis, we extracted data from the US National Health and Nutrition Examination Survey (NHANES) database, covering the years 2013 to 2018, focusing on individuals with recorded serum cotinine levels and infertility information. ETS exposure and fertility status were analyzed as independent and dependent variables, respectively. We applied weighted multivariate logistic regression method to evaluate the impact of ETS on infertility, including subgroup analyses for more detailed insights.

**Results:**

The study encompassed 3,343 participants. Logistic regression analysis revealed a notable positive correlation between ETS exposure and infertility, with an odds ratio (OR) of 1.64 (95% Confidence Interval [CI]: 1.14–2.36). We observed a non-linear relationship between ETS exposure and infertility risk. Notably, infertility risk increased by 64% in serum cotinine levels above 0.136 compared to that in serum cotinine levels below 0.011. Further, subgroup analysis and interaction tests showed consistent results across different segments, underscoring the robustness of the ETS-infertility link.

**Conclusion:**

Our findings suggest that environmental tobacco smoke exposure may be a contributing factor to infertility. These results reinforce the recommendation for women in their reproductive years to avoid ETS exposure, especially when planning for pregnancy.

## Introduction

Infertility, defined as the inability to conceive after at least 12 months of regular, unprotected sexual activity, reportedly affects approximately 8–12% of couples in their reproductive years worldwide (1, 2). In the United States, data shows an increase in infertility rates from 5.8% during 2006–2010 to 8.1% in 2017–2019 (3). Research by Zhou et al. highlights that in eight provinces and municipalities under China’s central government, 15.5% of women at risk of pregnancy experience infertility (2,680/17275). Notably, this rate rises to 25.0% (2,680/10742) among women actively trying to conceive, with a clear trend of increase alongside advancing age (4). Additionally, an upward trend in infertility rates is observed among women in the Middle East, North Africa, and South Asia (5). The underlying causes of infertility are diverse, including ovulatory dysfunction and tubal disease. Moreover, environmental elements like air pollution and endocrine-disrupting chemicals, combined with lifestyle factors such as obesity and tobacco use, are significant contributors to fertility issues (6, 7). Infertility mirrors various societal and health aspects, encompassing reproductive health, medical services, and living standards within a region or country. It has evolved into a significant concern for childbearing-age couples worldwide, markedly influencing women’s quality of life, mental health, and marital satisfaction, as recent studies indicate (8).

The widespread use of tobacco globally has positioned tobacco-related diseases as a principal public health concern. In 2020, it was estimated that tobacco smoking caused approximately 7.7 million deaths worldwide, with men accounting for 80% and current smokers for 87% of these fatalities. In affluent nations, tobacco-related deaths primarily stem from lung cancer, emphysema, heart attacks, strokes, and cancers of the upper gastrointestinal tract and bladder (9). Both firsthand and secondhand smoking, the latter also known as Environmental Tobacco Smoke (ETS) or passive smoking, pose significant health risks. ETS, a pollutant, emanates from exhaled smoke and burning tobacco products and comprises detrimental substances like nicotine, tar, carbon monoxide, and various carcinogens (10). Studies have linked ETS to various child health complications, including respiratory and perinatal disorders, and neurobehavioral issues, with passive smoking notably affecting children’s health (11). Furthermore, research has identified an association between elevated metal levels in children’s saliva and ETS exposure, indicating that ETS might increase the accumulation of harmful metals in children, thus augmenting health risks (12). Extended exposure to ETS has been linked to an increased risk of developing Chronic Obstructive Pulmonary Disease (COPD), especially in highly exposed residential and workplace environments (13).

Research has revealed a significant 54.9% increase in infertility risk among active smokers compared to non-smokers (14). The Anti-Müllerian Hormone (AMH), serving as an ovarian reserve marker, is observed to diminish in women who smoke, implying a detrimental effect on fertility (15). Conversely, the impact of passive second-hand smoke exposure on fertility remains ambiguous. Present epidemiological investigations into the correlation between ETS and infertility are sparse, underscoring the necessity for more extensive studies. This investigation delves into the potential association between ETS and infertility risk, employing data from the US National Health and Nutrition Examination Survey (NHANES). This study adopts a cross-sectional design to uncover crucial information that could aid in developing strategies to prevent infertility.

## Methods and materials

### Collection of research data

Data for this analysis were sourced from the US NHANES database, managed by the National Center for Health Statistics (NCHS). As a comprehensive and nationally representative survey program, NHANES collects diverse health-related data through methods including questionnaire-based interviews, physical examinations, laboratory tests, and more, utilizing a sophisticated multi-stage probability sampling design. The NCHS database is instrumental in advancing epidemiological and health sciences research. Specifically for this research, relevant NHANES survey data from 2013 to 2018 were meticulously gathered from the database, accessed through the official NHANES website.

### Study population

**Figure 1:**
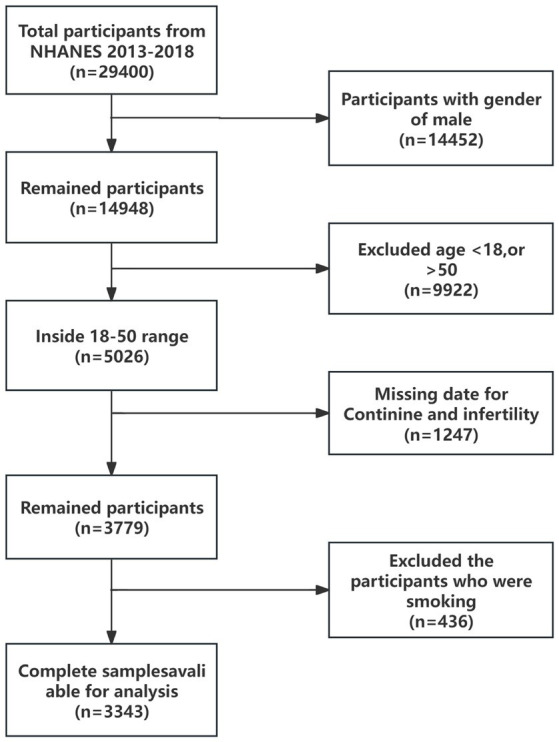
Flowchart of the participant selection process.

For this investigation, a selected portion of the NHANES data, spanning from 2013 to 2018, was scrutinized. This period was specifically chosen due to the inclusion of health questionnaires pertinent to infertility. Initially, the survey encompassed 29,400 adults. For our analysis, data from all male participants within this timeframe were excluded. The study then concentrated on female participants within the age range of 18 to 50 years. Records lacking serum cotinine concentration data were also disregarded. As a result, the analyzed sample ultimately consisted of 3,343 participants. Figure 1 provides a comprehensive depiction of the inclusion and exclusion criteria applied to the study population.

### Dependent and independent variables

In this study, infertility was defined following the 1995 guidelines of the World Health Organisation (WHO). According to this definition, infertility occurs in couples with a normal sexual life, where the male partner demonstrates normal reproductive function, and the couple has lived together for over a year without contraception, yet the female partner has not been able to conceive or maintain a pregnancy. To identify infertility cases, our research utilized self-reported information from the Women’s Reproductive Health Questionnaire within the NHANES database, focusing on the variable RHQ074. When participants answered “Yes” to the question “Tried for a year to become pregnant?,” it was interpreted as indicative of infertility, whereas a “No” answer implied fertility.

In the present study, ETS, constituting exhaled smoke from smokers and that produced by cigarette combustion, was identified as the independent variable. Its quantification was based on serum cotinine concentrations. For analysis purposes, ETS exposure was stratified into three groups according to serum cotinine levels: Group T1, with levels below 0.011 ng/mL; Group T2, encompassing levels from 0.011 to 0.136 ng/mL; and Group T3, levels above 0.136 ng/mL.

### Concomitant variable

Drawing on insights from related studies, this research extended its analysis to include various covariates in addition to ETS, aiming to control for potential confounding variables. Selected demographic covariates comprised age (ranging from 18 to 50 years), race (categories included non-Hispanic white, non-Hispanic black, non-Hispanic Asian, Mexican American, other Hispanic, and other races), marital status (encompassing married, divorced, separated, widowed, cohabitating, and never married), and poverty-to-income ratios, all sourced from the NHANES database. In this study, physical health parameters including Body Mass Index (BMI), sedentary lifestyle, and waist circumference measurements were factored in. This inclusion is based on existing research which suggests that lifestyle factors, especially inadequate physical activity, can potentially influence fertility either directly or indirectly. Furthermore, the occurrence of two chronic diseases, hypertension and diabetes, was factored into the analysis. The integration and scrutiny of these covariates were intended to enhance the accuracy of evaluating the correlation between ETS exposure and infertility in the NHANES population.

### Statistical analysis

In this research, to account for complex sampling design, data in this study were weighted using appropriate sample weights provided by NHANES. Baseline population characterization was achieved using descriptive statistics, which involved calculating means and standard deviations for continuous variables, and identifying percentages for categorical variables. The association between EST and infertility was explored through multivariate logistic regression analysis. This analysis incorporated three models: Model 1, an unadjusted basic model; Model 2 adjusted for key demographic factors such as age, race, marital status, and the ratio of poverty to income; and Model 3, which comprehensively adjusted for all covariates. Furthermore, to determine their effect on the association between ETS and infertility, weighted univariate logistic regression analyses were performed on these variables. All statistical evaluations were carried out using R software version 4.3.2 (available at http://www.R-project.org), with statistical significance set at a two-sided *p*-value of less than 0.05.

## Results

### Baseline characteristics

Table 1 presents the demographic and clinical characteristics of the study’s 3,343 participants, comprising 2,978 individuals without infertility and 365 with infertility. The average age of the participants was 33.7 years. Notably, there was a significant age between those with infertility (average age 37.2 years) and those without (average age 33.2 years), suggesting an increased incidence of infertility in older individuals. Marital status appeared to influence infertility prevalence, being higher in married (61.77%) and divorced (10.25%) participants compared to their unmarried counterparts (44.00% and 7.78%, respectively). Additionally, the infertility group demonstrated a higher average BMI of 31.7 and waist circumference of 101.6 cm, versus 29.5 and 95.4 cm, respectively, in the non-infertility group, indicating a correlation between obesity and infertility. Alcohol consumption was also more prevalent among the infertility group (10.93% vs. 7.54%). Comorbid conditions such as hypertension (23.84% vs. 15.92%) and diabetes mellitus (10.41% vs. 4.94%) were notably higher in the group experiencing infertility. A significant observation was the marked difference in average serum cotinine levels. Individuals in the infertility group exhibited an elevated average of 49.7 ng/mL, while the average in the non-infertility group was 37.6 ng/mL. This suggests a greater exposure to secondhand tobacco smoke among those struggling with infertility.

**Table 1:** Baseline characteristics of participants (*N* = 3,343).

	All women (*N* = 3,343)	Infertility (*N* = 365)	Non-infertility (*N* = 2,978)	*p*-value
Age (year)	33.7	37.2	33.2	<0.0001^****^
18–29	37.7%	73 (20.00%)	1,186 (39.83%)	
30–39	28.5%	131 (35.89%)	822 (27.60%)	
≥40	33.8%	161 (44.11%)	970 (32.57%)	
Race				0.0302^*^
Mexican	17.8%	53 (14.52%)	544 (18.27%)	
Other Hispanic	10.8%	35 (9.59%)	326 (10.95%)	
White	32.2%	144 (39.45%)	933 (31.33%)	
Black	21.5%	71 (19.45%)	648 (21.76%)	
Asian	12.7%	47 (12.88%)	376 (12.63%)	
Other race	5.0%	15 (4.11%)	151 (5.07%)	
Marital Status				<0.0001^****^
Married	43.4%	223 (61.77%)	1,177 (44.00%)	
Widowed	2.4%	2 (0.55%)	30 (1.12%)	
Divorced	8.8%	37 (10.25%)	208 (7.78%)	
Separated	5.3%	15 (4.16%)	112 (4.19%)	
Never married	26.6%	45 (12.47%)	794 (29.68%)	
Living with partner	13.5%	39 (10.80%)	354 (13.23%)	
Ratio of family income				0.0008^***^
≤1.85	44.3%	138 (40.71%)	1,343 (49.52%)	
>1.85	55.7%	201 (59.29%)	1,369 (50.48%)	
Hypertension				<0.0001^****^
Yes	16.8%	87 (23.84%)	474 (15.92%)	
No	83.2%	278 (76.16%)	2,504 (84.08%)	
Diabetes				<0.0001^****^
Yes	5.5%	38 (10.41%)	147 (4.94%)	
No	62.8%	317 (86.85%)	2,784 (93.49%)	
Borderline	31.7%	10 (2.74%)	47 (1.58%)	
Sedentary time (min)	418.3	409.2	419.4	0.1418
<300	48.1%	170 (46.70%)	1,436 (48.29%)	
300–600	41.7%	152 (41.76%)	1,242 (41.76%)	
>600	10.2%	42 (11.54%)	296 (9.95%)	
BMI	29.7	31.7	29.5	<0.0001^****^
0–25	34.1%	98 (27.22%)	1,043 (35.36%)	
25–30	23.6%	70 (19.44%)	720 (24.41%)	
>30	42.3%	192 (53.33%)	1,187 (40.24%)	
Waist (cm)	96.1	101.6	95.4	<0.0001^****^
<70	4.0%	7 (2.02%)	127 (4.40%)	
70–120	81.5%	279 (80.40%)	2,446 (84.75%)	
>120	14.5%	61 (17.58%)	313 (10.85%)	
Drinking				0.0097^**^
Yes	6.4%	34 (10.93%)	180 (7.54%)	
No	93.6	277 (89.07%)	2,206 (92.46%)	
Continine (ng/mL)	38.9	49.7	37.6	0.0167^*^
T1	33.1%	117 (32.05%)	988 (33.18%)	
T2	33.6%	115 (31.51%)	1,008 (33.85%)	
T3	33.3%	133 (36.44%)	982 (32.98%)	

^*^p < 0.05, ^**^p < 0.01, ^***^p < 0.001, ^****^p < 0.0001.

### Multiple regression model results

**Figure 2:**
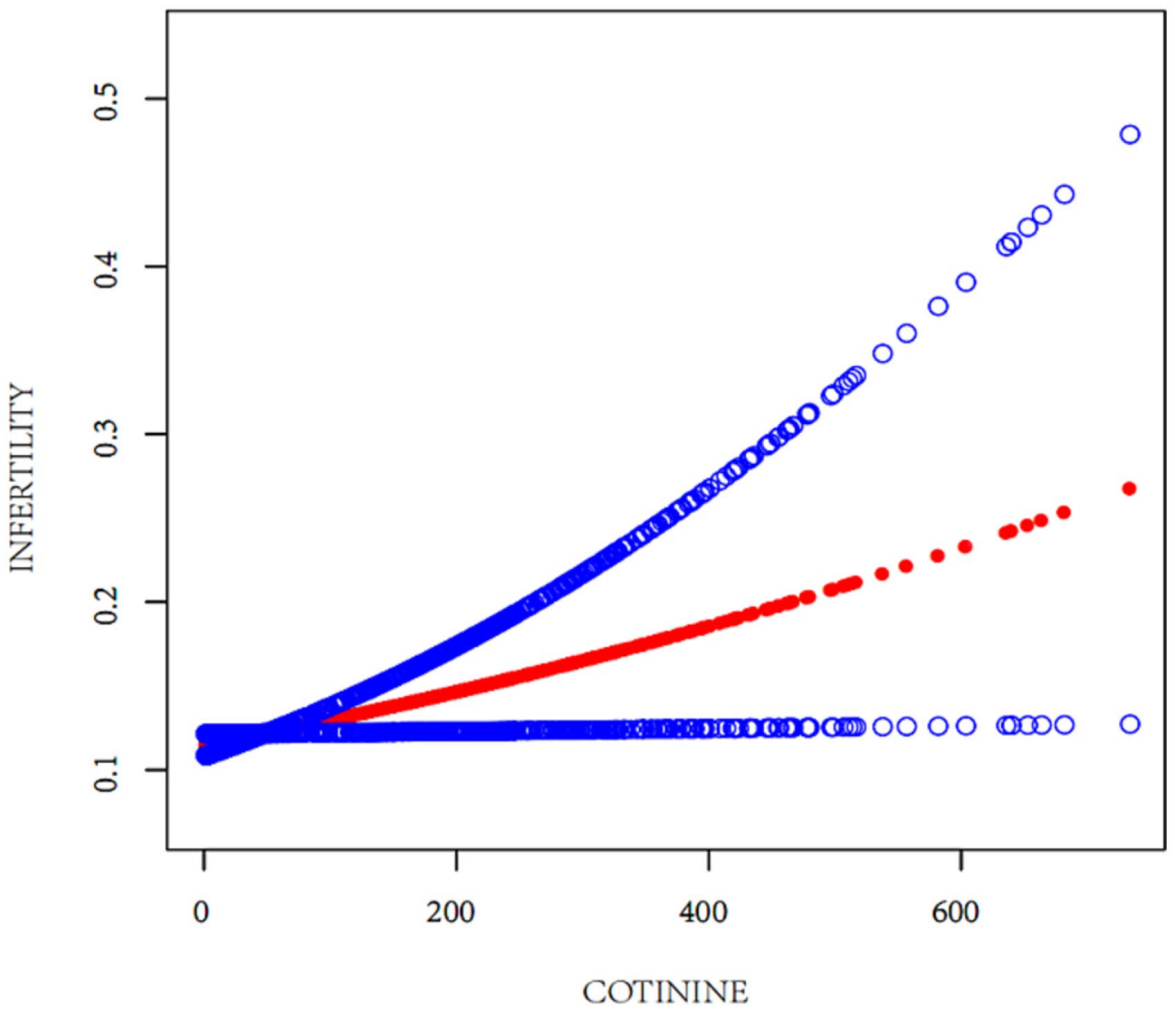
The correlation between cotinine and infertility rate.

The relationship between EST and infertility was examined using multifactorial logistic regression analysis. As depicted in Table 2, a significant increase in infertility prevalence was associated with elevated serum cotinine levels. This relationship was methodically analyzed through the development of three distinct models. Notably, Model 3 demonstrated a statistically significant and positive association between serum cotinine concentration and infertility, taking into account all pertinent confounders. Specifically, for serum cotinine concentrations exceeding 0.136 ng/mL, there was a 64% increase in infertility risk for every incremental increase in cotinine level. The regression curve, illustrated in Figure 2, reinforces the positive correlation between infertility risk and serum cotinine levels, suggesting a substantial association between higher exposure to environmental tobacco smoke, as indicated by serum cotinine, and an increased prevalence of infertility.

**Table 2:** Multivariate logistics regression model of continine infection and infertility.

	Model 1		Model 2		Model 3	
	OR (95%Cl)	*p-*value	OR (95%Cl)	*p-*value	OR (95%Cl)	*p-*value
Continine (ng/mL)						
T1 (<0.011)	1.0		1.0		1.0	
T2 (0.011–0.136)	0.96 (0.73, 1.26)	0.7881	1.07 (0.80, 1.44)	0.6563	1.05 (0.75, 1.48)	0.7663
T3 (>0.136)	1.14 (0.88, 1.49)	0.3182	1.37 (1.00, 1.88)	0.0476*	1.64 (1.14, 2.36)	0.0073**
*P* for trend	1.00 (1.00, 1.01)	0.1857	1.01 (1.00, 1.01)	0.0397*	1.01 (1.00, 1.02)	0.0028**

^*^p < 0.05, _**_p < 0.01. To examine the link between EST and infertility, complex multivariate logistic regression analysis was conducted, considering a range of potential influencing factors. The analysis included Model 1, which did not account for any confounding variables, and Model 2, which incorporated adjustments for age, marital status, the ratio of poverty-to-income, and racial background of the participants; and Model 3, which extended these adjustments to encompass all covariates.

### Subgroup analysis

Further, Subgroup analyses and interaction tests were employed to investigate the consistency in the link between EST and infertility across diverse demographic and clinical factors. The data were segmented based on several variables: age, ethnicity, marital status, alcohol use, sedentary time, BMI, waist size, and the presence or absence of chronic diseases such as hypertension and diabetes. This approach facilitated a comprehensive exploration of how these elements might influence the relationship between secondhand smoke exposure and the likelihood of infertility.

**Figure 3:**
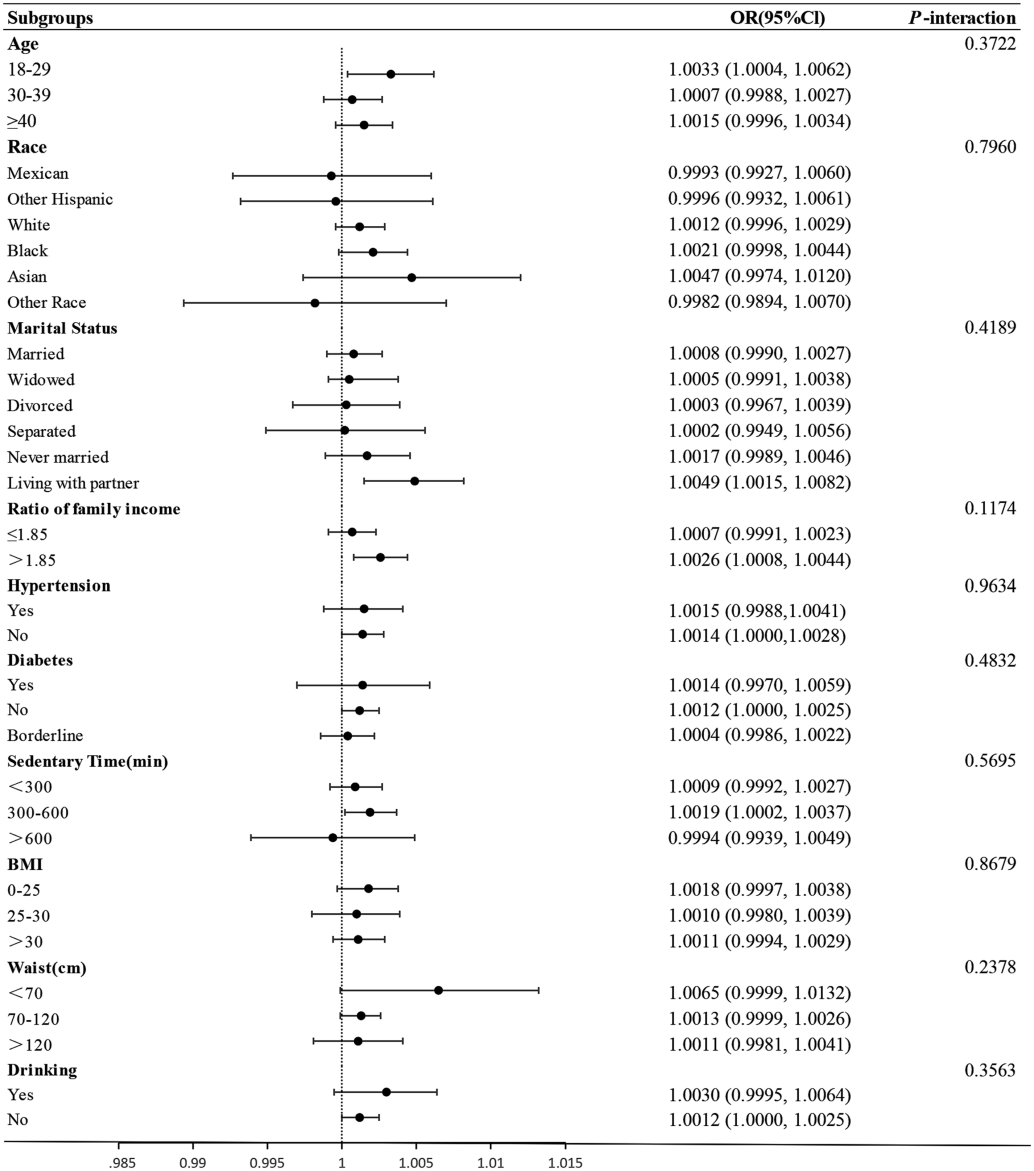
The forest of subgroup analyses of the effect of ETS on infertility.

The forest plot in Figure 3 reveals no significant interaction between ETS and any stratification factors, as evidenced by all *p*-values >0.05. Specifically, the interaction values were 0.3722, 0.7960, 0.4189, 0.1174, 0.9634, 0.4832, 0.5695, 0.8679, 0.2378, and 0.3563. These findings suggest a uniform association between ETS and infertility across all examined subgroups.

## Discussion

Infertility, clinically defined as the inability to conceive without contraception for a period exceeding 1 year, employs this criterion as a diagnostic benchmark. The present study included female patients with infertility, identified through the RHQ074 questionnaire from the NHANES, ensuring an objective delineation of the infertility cohort (16, 17). Cotinine, the predominant metabolite of nicotine, stands as the paramount biomarker for discerning exposure among smokers and nonsmokers to ETS (18, 19). Consequently, we utilized serum cotinine levels as a quantitative measure for assessing ETS exposure, post exclusion of actively smoking females. The aim of our investigation was to examine the correlation between ETS exposure and infertility. Our analysis revealed that exposure to ETS significantly increased the risk of infertility in women. Notably, serum cotinine levels above 0.136 ng/mL were linked to a 64% heightened risk of infertility for each incremental increase in cotinine concentration.

Numerous studies have highlighted the impact of environmental contaminants like heavy metals, organic solvents, pesticides, and endocrine-disrupting chemicals on female reproductive health. These pollutants have been implicated in causing infertility and negatively affecting pregnancy outcomes (20). However, the link between ETS and infertility has not been extensively studied (21). A prior study, using follicular fluid cotinine concentrations to measure exposure, did not find a significant variation in the rates of pregnancy and fertilization among active smokers, passive smokers, and non-smokers. However, it’s important to note that the sample size of this study was somewhat limited (*N* = 197; 103 active smokers; 26 passive smokers) (22). Research by Wesselink revealed that women who smoked more than 10 cigarettes daily for over a decade had lower fertility rates compared to never-smokers, while the effect of male smoking and passive smoking on fertility was less pronounced (23). Other studies have demonstrated a negative impact of ETS exposure on fertility. A retrospective study among women of childbearing age indicated a clear statistical increase in delayed conception for those exposed to secondhand smoke. The odds ratio was 1.17 [95% CI 1.02–1.37] for exposure lasting over 6 months, and 1.14 [95% CI 0.92–1.42] for exposure extending beyond 12 months (24). Peppone et al. found that ETS exposure led to higher difficulties in pregnancy and increased fetal loss rates compared to those unexposed (25). In a study by Andrew Hyland involving 93,679 postmenopausal participants from the Women’s Health Initiative Observational Study (WHI OS), it was discovered that never-smokers with high lifetime secondhand smoke (SHS) exposure experienced more pregnancy losses than those unexposed, with adjusted ORs of 1.18 (95% CI 1.02 to 1.35) for infertility and 1.18 (95% CI 1.06 to 1.31) for earlier onset of menopause (26). A retrospective analysis of a prospective cohort study revealed a marked elevation in the risk of implantation failure during pregnancy in women subjected to STS with an OR of 1.52 (95% CI = 1.20–1.92) and a relative risk (RR) of 1.17 (95% CI = 1.10–1.25) (27). A recent systematic review encompassing six studies concluded that exposure to secondhand smoke adversely affects fertility rates (28). Additionally, it has been observed that tobacco smoke exposure during gestation, as well as in young girls either during childhood or *in utero* has been linked to reduced fertility in offspring and an increased risk of spontaneous abortion in adulthood, respectively (29, 30). Our study found a correlation between ETS exposure and infertility, with a non-linear relationship. Subgroup analyses were performed to control for confounding factors, confirming that no included factors significantly interacted with ETS.

Research on the impact of ETS on infertility is sparse. Exposure to ETS may disrupt women’s endocrine hormone balance, potentially leading to infertility. Elevated prolactin levels, a condition known as hyperprolactinemia, have been implicated in infertility in some women. In a study of 314 women undergoing *in vitro* fertilization (IVF), those exposed to ETS exhibited significantly higher prolactin levels compared to unexposed non-smokers (31). Additionally, non-smoking Chinese women exposed to ETS showed decreased urinary estrone conjugate (E1C) during unsuccessful conception attempts (32). Increased follicle-stimulating hormone (FSH) concentrations were found to be correlated with both active and passive smoking in women aged 38–49 (33). ETS also adversely impacts mental health during both natural and assisted reproductive processes, with varying effects across different pregnancy stages (34). Egg quality, influenced by its follicular microenvironment, is susceptible to a range of physiological, biochemical, and environmental factors, including tobacco smoke, which may cause follicular damage and oocyte dysfunction (35, 36). Cigarette smoke components contribute to follicular lipid peroxidation and DNA damage, as evidenced in animal studies showing its effects on oocyte depletion, apoptosis, and oxidative stress, leading to fewer ovulating follicles (37). A recent investigation found that ETS might impact assisted reproduction outcomes by affecting oocyte availability, but oxidative stress did not mediate these effects (38). Smoking-related DNA methylation changes have also been linked to infertility (39). Finally, passive smoking detrimentally affects embryo quality, leading to a higher prevalence of lower-grade embryos among women exposed to cigarette smoke (40).

This study demonstrates significant strengths. It comprises a detailed cross-sectional analysis of women throughout the United States, establishing for the first time a correlation between infertility and ETS. The logistic regression model applied in this study investigates the complex relationship between infertility and ETS, yielding insights of higher clinical relevance than previous research. By analyzing data from NHANES, which reflects the diverse demographic of the nation, enables the potential application of our findings to a wider population of childbearing-aged women in the United States. Furthermore, the discoveries made through this research could be instrumental in developing strategies to improve infertility treatments and enhance the understanding of women’s reproductive health.

However, this study has certain limitations. First, the applicability of these findings to women outside the United States might be limited. Moreover, the cross-sectional design of our study limits our ability to infer a direct cause-and-effect relationship between infertility and exposure to ETS. Therefore, future prospective studies with larger sample sizes and more rigorous clinical designs are needed to further investigate the association between ETS exposure and infertility. Finally, on the one hand, the investigated participants with missing data on serum cotinine concentrations were excluded from this study, and on the other hand, when performing the subgroup analyses, we did not include in the analyses the history of infertility among family, which made it impossible to assess whether the part of the participants with missing data had an impact on the results of this study.

## Conclusion

In this study, we discovered a potential association between infertility and exposure to Environmental Tobacco Smoke. Notably, we found that infertility risk increased by 64% with every unit increase in serum cotinine levels above 0.136 ng/mL. This finding highlights the importance of ETS avoidance in the context of female reproductive health. We suggest that our study provides strong support for recommending ETS avoidance among women preparing for pregnancy, which could also positively impact socio-demographic factors. However, to validate these observations, further comprehensive prospective studies are still necessary.

## Data availability statement

The original contributions presented in the study are included in the article/supplementary material, further inquiries can be directed to the corresponding author.

## Author contributions

LP: Conceptualization, Formal analysis, Investigation, Writing – original draft. XL: Methodology, Writing – review & editing. BC: Formal analysis, Investigation, Writing – review & editing. XW: Supervision, Writing – original draft.
